# Allelochemical, Eudesmane-Type Sesquiterpenoids from *Inula falconeri*

**DOI:** 10.3390/molecules15031554

**Published:** 2010-03-10

**Authors:** Abdul Latif Khan, Javid Hussain, Muhammad Hamayun, Sang-Mo Kang, Hak-Youn Kim, Kazuo N. Watanabe, In-Jung Lee

**Affiliations:** 1 School of Plant Biosciences, College of Agriculture and Life Sciences, Kyungpook National University, Daegu, Korea; 2 Kohat University of Science & Technology, Kohat 26000, NWFP, Pakistan; 3 Gene Research Center, University of Tsukuba, Ibaraki, Japan; 4 Department of Environmental Conservation and Disaster Prevention, College of Environment, Keimyung University, Daegu, Korea

**Keywords:** *Inula falconeri*, eudesmane sesquiterpenes, allelopathic effect

## Abstract

We have identified through bioassay guided isolation an allelochemical, eudesmane-type sesquiterpeniod, 3β-caffeoxyl-β1,8α-dihydroxyeudesm-4(15)-ene (**1**), from an endemic plant species growing in the Himalayas. In our search for the bioactive subfraction, the hexane one was highly significant, showing 100% inhibition of lettuce seed growth at 100 ppm while other subfractions (chloroform, ethyl acetate, butanol and water) exhibited inhibitory to stimulatory allelopathic effects. The bioactive hexane subfraction was subjected to chromatographic techniques, using lettuce seeds (*Lactuca sativa*) as indicator species to reveal the bioactive allelopathic fraction. This resulted in the isolation of compound **1**, whose structure was elucidated through NMR techniques*.* The compound presented 92.34% inhibitory effect on the growth of lettuce at 500 ppm. Further field level experiments may help develop an environmentally friendly herbicide from this lead.

## 1. Introduction

Allelochemicals are secondary metabolites released by plant species in their environment. They play a pivotal role in agricultural and functional ecology [1,2]. Identification of such chemical constituents has created a thrust to address several problems confronted in plant physiology, pathology, environment and crop production. Pursuing and developing natural products as environmentally friendly herbicides can eliminate damages to human health as well as to ecosystem. Efforts have been made to elaborate the allelopathic effects of various plant species. Genus *Inula* is famous for its diverse biological activities *i.e.,* anticancer, antibacterial, hepaprotective, cytotoxic, and anti-inflammatory, however limited information is available on their role in allelopathy [3]. This is the first report to elucidate an allelopathic interaction of *Inula falconeri* from the genus. *I. falconeri* was reported as a potential allelopathic plant species among other four plants while investigating the role of method and concentration in allelopathy [2]. *I. falconeri* is endemic to the Himalayas and distributed in the northern areas of Pakistan and western Tibet [4]. It grows at 2,400 m altitude, however, it has also been observed at 3,000 m. At lower altitudes, it is found mainly near agricultural fields and household gardens. Due to its colorful flowers, it has been frequently used for decoration purposes. Local people revealed that *I. falconeri* was abundant at lower altitudes but due to over exploitation the plant population has been reduced considerably over the years as the plant was eradicated as an unwanted species near or in agricultural fields [5]. The aim of this study was to explore and identify the allelopathic potential of this plant species by isolating and characterizing potential allelochemicals through a bioassay guided approach.

## 2. Results and Discussion

**Figure 1:**
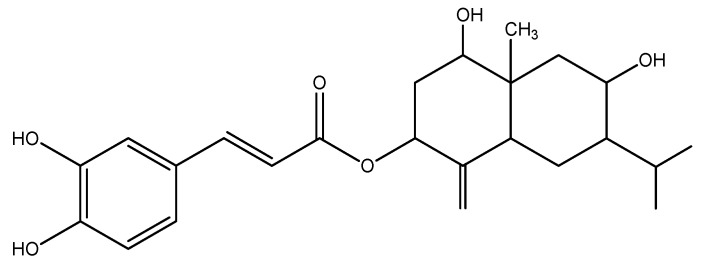
Structure of compound **1**.

The structure of compound **1** (Figure 1) was established conclusively by UV, IR, MS and extensive ^1^H- and ^13^C-NMR spectra analysis and comparison with a published report – identifying it as a known eudesmane-type sesquiterpene (3β-caffeoxyl-1β,8α-dihydroxyeudesm-4(15)-ene) [9].

**Figure 2:**
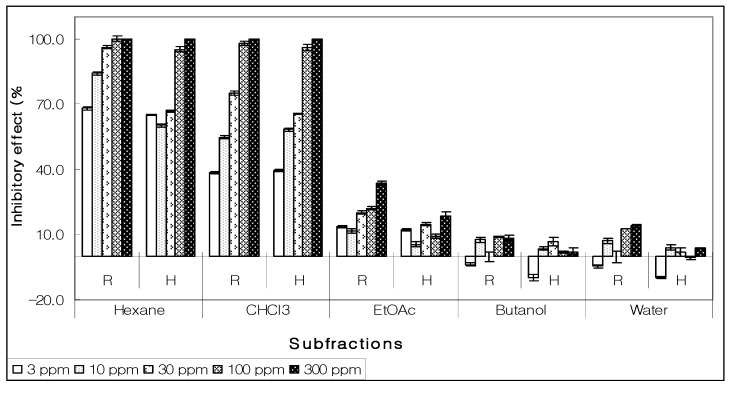
Allelopathic effect of various subfractions on growth of root (R) and hypocotyls (H) of lettuce seeds at different concentrations.

Pursuing the active allelopathic subfraction after solvent-solvent partition, the hexane subfraction was seen to inhibit the growth of root and hypocotyls of lettuce seedlings. At lower concentrations, it presented higher specific activity *i.e.,* inhibiting 50% growth at 3 ppm and at maximum concentration (300 ppm), no seeds germinated [5]. Chloroform subfraction revealed a dose dependent effect on lettuce seed’s growth; however, the effect was insignificant compared to that of hexane subfraction. Other subfractions (ethyl acetate, butanol and water) exhibited no EC_50_ activity and their allelopathic effects ranged from insignificantly inhibitory to stimulatory (Figure 2) [5].

**Figure 3:**
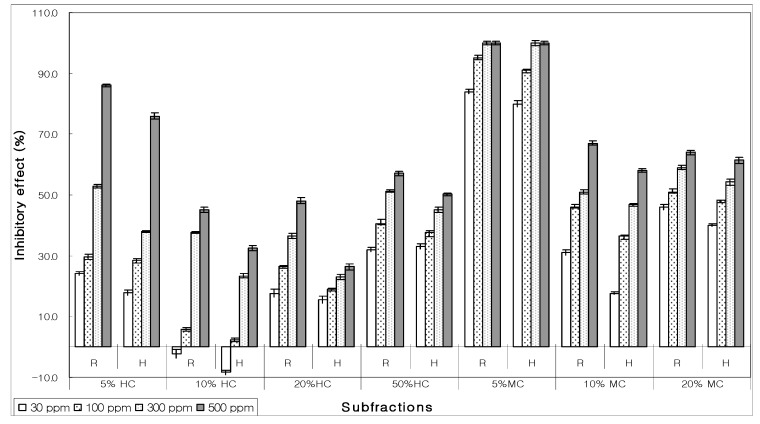
Allelopathic behavior of various subfractions on the growth of root (R) and hypocotyls (H) of lettuce seed derived from bioactive hexane extract using silica gel column chromatography (HC – hexane in chloroform; MC – methanol in chloroform.)

After column chromatography of bioactive hexane fraction under different solvent regimes, seven subfractions were assayed for their allelopathic behavior. Most subfractions exhibited moderate to high inhibition at maximum concentration (500 ppm) but at lower concentration (30 ppm) none of the subfractions had EC_50_ value except 5% MeOH-CHCl_3_. It inhibited lettuce seed germinations at 300 ppm and shown EC_50_ activity at 30 ppm (Figure 3).

**Figure 4:**
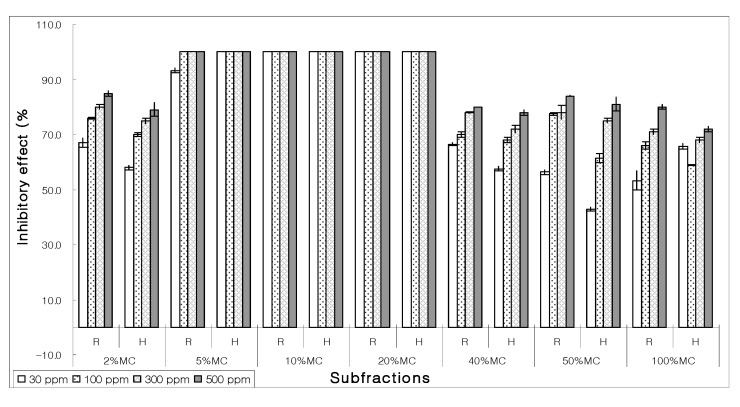
Pattern of allelopathic effect on the growth of root (R) and hypocotyls (H) of bioactive 5% methanol: chloroform (MC) using a silica gel column equipped with an automated fractionator.

The bioactive 5% MeOH-CHCl_3_ was further fractionated. The resultant seven subfractions were bio-assayed for their EC_50_ activity. Nearly every subfraction presented moderate suppression; however, 5%, 10%, and 20% MeOH-CHCl_3_ subfractions had 100% inhibitory effect toward germination of lettuce seeds on all concentrations (Figure 4).

**Figure 5:**
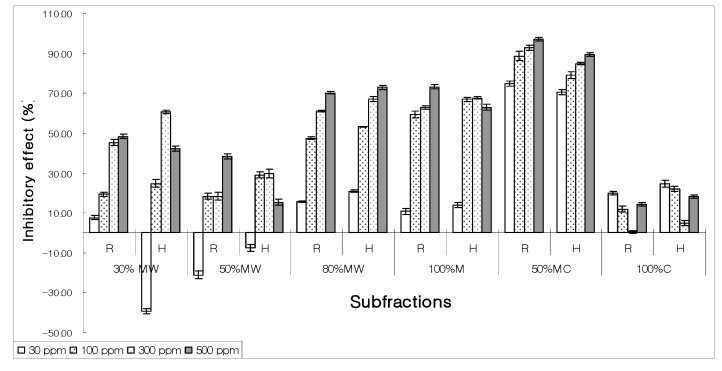
Allelopathic effect of different subfractions of bioactive 5%, 10% and 20% MeOH-CHCl_3_ on the growth of root (R) and hypocotyls (H) of lettuce seeds after preparative reverse phase solid-phase extraction. (MC – methanol in chloroform; MW – methanol in water; M – methanol; C – chloroform.

Due to similar allelopathic effect, 5%, 10%, and 20% MeOH-CHCl_3_ fractions were combined and subjected to reverse-phase chromatography, which afforded six new subfractions. Among these, 50% MeOH-CHCl_3_ exhibited a significant inhibitory effect. All concentrations of this bioactive subfraction were more than 70% inhibitory towards growth of lettuce seeds (Figure 5).

**Figure 6:**
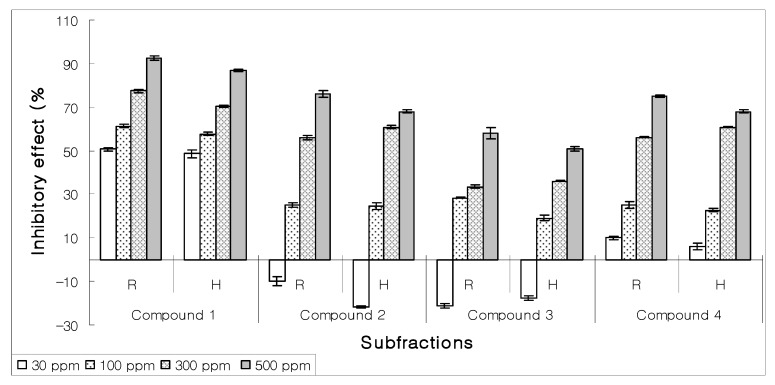
Effect on the growth of lettuce seeds of four purified compounds from 50% methanol: chloroform subfraction using RP-HPLC.

**Figure 7:**
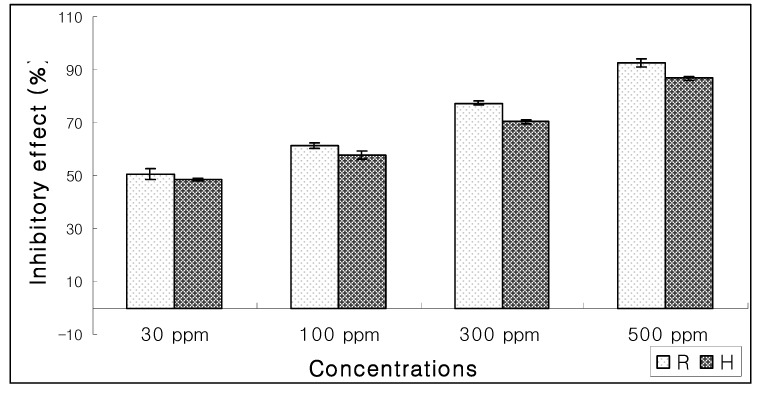
Inhibitory effect of compound (**1**) on the growth of root (R) and hypocotyls (H) of lettuce.

Four compounds purified through RP-HPLC were assayed for their EC_50_ (Figure 6). Compound **1** exhibited significant inhibitory effect (92.34%) towards the growth of lettuce seeds at 500 ppm while EC_50_ activity was shown at 30 ppm concentration (Figure 7). Due to paucity of material the other three compounds could not be identified.

Earlier *I. falconeri* was screened among four plant species for allelopathic potential of leaf leachates [2]. The hexane subfraction of *I. falconeri* was revealed as the most potent, not only exhibiting allelopathic effects but also displaying a significant zone of inhibition against *Alternaria alternata* and *Rhizoctonia solani* and an insignificant effect against *Fusarium oxysperum* [5]*.* Previous reports on *I. falconeri* established the presence of phenolic acids (caffeic acid and chlorogenic acid) and flavonoids (rutin, quercetin and kaempferol) [4], which have already been proved to display allelopathic effects [6,7,8]. Besides broader biological effects of various classes of sesquiterpenoids, these have also been reported to carry allelopathic potential [10,14].

Compound **1** has already been reported to have antibacterial properties against human pathogens [9]. Although eudesmane-type sesquiterpenes have been reported for their cytotoxicity, enzyme inhibitory and antimicrobial effects [9,10,11] however, it is now also reported as a potent allelochemical.

## 3. Experimental

### 3.1. General

The ^1^H and ^13^C NMR, COSY, HMQC and HMBC spectra were recorded in CD_3_OD using TMS as internal standard on a Bruker spectrometer operating 500/125 MHz. The chemical shift values are reported in ppm (*δ* ) units and coupling constant (*J*) in Hz. EI, CI MS were recorded using JMS-HX-110 with data acquisition system and on JMS-DA 500 mass spectrometer. HPLC (TOSOH, Tokyo, Japan; UV PD 8020) was performed equipped with a reversed-phase column (Inertsil1 ODS-3, 10 × 250 mm, GL Sciences Inc., Tokyo). Methanol and milli-Q water were used as elution solvents in HPLC with a flow rate of 2.8 mL/min.

### 3.2. Plant Material

*I. falconeri* was collected from Deosai Plains Skardu (35º03′23.26″N-75º27′39.69″E) in Himalayan regions of the Northern Areas of Pakistan in August 2007—flowering season of the plant. It was identified by plant taxonomists at the Department of Botany, Kohat University of Science and Technology, Kohat with herbarium number If-337K.

### 3.3. Extraction and Isolation

Dried plant material of *I. falconeri* (1.20 kg) was soaked in pure MeOH (2.5 L) and extracted for 30 days at 5 ºC in dark. MeOH extract was evaporated to dryness *in vacuo*. It was subjected to solvent-solvent partition into hexane, chloroform, ethyl acetate, butanol and water subfractions. All subfractions were screened for their allelopathic effect. The hexane subfraction revealed higher EC_50_ activity. This subfraction (8 g) was extracted five times with hexane to get polar compounds. It was later subjected to column chromatography using silica gel-60 (Wako Pure Japan; 19 mm i.d. 300 mm) in successive elution with hexane-CHCl_3_ (5%, 10%, 20% and 50%) and CHCl_3_-MeOH (5%, 10% and 20%) (300 mL each).

After EC_50_ activity and TLC analysis, the bioactive 5% CHCl_3_ in MeOH fraction (980 mg) was selected for column chromatography using silica gel (1cm i.d., 40cm i.l; Kiriyama Japan) on a SF-2120 fractionator (Advantech Japan). Elution with MeOH-CHCl_3_ (2%, 5%, 10%, 20%, 40%, 50% and 100%) was used. Looking for significant allelopathic fraction and then analyzing the TLC spots of each subfraction, the bioactive 5%, 10% and 20% MeOH-CHCl_3_ fractions (278 mg) were combined and subjected to preparative reverse phase solid-phase extraction (Waters C18 Sep-Pak), eluting successively with 30%, 50%, and 80% MeOH-H_2_O and 100% MeOH, 50% MeOH-CHCl_3_ and 100% CHCl_3_ (100 mL each).

The bioactive 50% MeOH-CHCl_3_ subfraction (39 mg) were subjected to RP-HPLC using 50% and 80% methanol in water and 100% methanol. Four compounds **1–4** were isolated. Upon testing the EC_50_ activity of the pure constituents, the most active allelochemical, compound **1** (2.87 mg) with Rt 23.35–25.12 min was obtained and its structure determined as 3β-caffeoxyl-1β,8α-dihydroxyeudesm-4(15)-ene (an eudesmane-type sesquiterpene) [9] (Figure 1).

### 3.4. Allelopathic Effects

The allelopathic potential of each fraction isolated from chromatographic column was assayed [12,13]. Lettuce seeds (*Lectuca sativa*, Takii Seed Co. Ltd., Japan) were used as indicator species to know the effective concentration of extract/compound that induces 50% inhibition of tested organism (EC_50_) when exposed to various concentrations of the fractions [12]. The inhibitory effects of fractions were studied by the specific activity method. Five concentrations *viz*. 3, 10, 30, 100 and 300 ppm of hexane, chloroform, ethyl acetate, butanol and water subfractions were used for EC_50_ bioassay. Four concentrations *i.e.*, 30, 100, 300 and 500 ppm of every successive subfraction of the bioactive hexane subfraction were then used for the EC_50_ bioassay. The concentrations were dissolved in 2% DMSO before application to the lettuce seeds. Initial concentration was 1,000 ppm. A filter paper (27 mm, Type Roshi Kaisha, Tokyo) was placed in a glass Petri dish. The dilutions were applied on filter paper and thus allowed to spread over it. Seven lettuce seeds were placed on it and dishes were sealed and packed for incubation for 72 hours at room temperature. For each fraction, mean, SD variance [13] and standard error were calculated to determine inhibition pattern at various concentration levels. While using EC_50_, specific activity of each extract was determined. Specific activity of subfractions was measured using specific activity = 1/EC_50_ × Concentration [12].

## 4. Conclusions

Bioassay guided investigations from *I. falconeri* resulted in purification of an allelochemical, 3β-caffeoxyl-1β,8α-dihydroxyeudesm-4(15)-ene (**1**) —an eudesmane-type sesquiterpene. The compound showed 92.34% inhibitory effect towards the growth of root and hypocotyls of lettuce seeds.
